# Influence of crack width on carbonation depths in functionally layered concrete

**DOI:** 10.1617/s11527-025-02643-8

**Published:** 2025-04-28

**Authors:** Jessica C. Forsdyke, Janet M. Lees

**Affiliations:** Department of Engineering, University of Cambridge, 7a JJ Thomson Ave, Cambridge, CB3 0FA UK

**Keywords:** Durability, Functionally layered concrete, Accelerated carbonation, Cracking

## Abstract

In functionally graded concrete, an outer durable concrete layer with higher cement can be used to protect lower grade interior concrete to maintain durability performance while reducing embodied carbon. However, flexural cracks in reinforced functionally layered beams can penetrate through the durability layer into interior concrete. Accelerated carbonation behaviour of cracked beams with different durability layer thicknesses and crack widths was investigated. Digital tools were used to trace the carbonation profiles. In regions remote from cracks the durability layer reduced carbonation ingress. At a crack, the carbonation front in the layered beams progressed into the interior mix. The width of the carbonation influence zone around the reinforcement was smaller when the steel was fully encased in the durability layer.

## Introduction

Functionally graded concrete is a promising route for the reduction in overall cement consumption of concrete structures [1]. To improve the efficiency of cement use, concrete mixes with high cementitious content are placed only in areas where they are required. One application of functional grading is through the use of two or more horizontal concrete layers within a single element. Such structures can be designed for durability purposes whereby an outer high performance durability layer protects embedded steel in reinforced concrete by preventing the ingress of aggressive substances. Since the majority of durability concerns primarily affect the outer surface of concrete elements, the high durability mix is often not required within the bulk of the element, and less CO$_2$ intensive mixes can be utilised there to improve overall material efficiency.

Maalej and Li first proposed the use of functionally graded concrete beams to improve durability [2]. Subsequent studies found that a durable layer of fibre reinforced [3–6] or ultra-high-toughness [7] cementitious composite surrounding the longitudinal reinforcement led to a better resistance to steel corrosion and reduced crack openings under load. Wen et al. [8] investigated the effect of adding low permeability ‘durability’ layers of varying thicknesses to reinforced concrete to protect steel reinforcement from corrosion. Papadakis et al. [9] found that cement-lime mortar coatings on the concrete surface were effective in postponing the onset of carbonation induced corrosion. Forsdyke and Lees [10] determined that an outer higher cement durability layer provided an effective means to delay the carbonation front into a lower cement inner concrete layer. These studies suggest that durability layers hold promise. However, one potential issue is whether cracks that propagate through a durability layer can undermine the durability performance. Of particular interest are the ramifications associated with the progression of carbonation since carbonation reduces the pH in concrete and facilitates the corrosion of steel reinforcement.

The interaction between flexural cracking and carbonation in functionally layered concrete members is unknown. The presence of an outer durability layer with higher ductility may reduce the crack width for a given load, which could be beneficial if the carbonation progression is crack width dependent. However, flexural tensile cracks through the outer durability layer could provide an undesirable route for carbonation to extend into the lower resistance interior bulk concrete. This points to a need to better understand carbonation of functionally layered concrete under loaded conditions, as this is representative of structures in practice.

The carbonation behaviour of cracked functionally layered reinforced beams is investigated in the current work to gain new insights into the impact of different durability layer thicknesses and crack widths on the carbonation progression. Conventional concrete mixes with aggregates are used within the layers to mimic compositions that can be rapidly adopted into practice. Digital image processing techniques are applied to discretise the tortuous carbonation fronts observed in cracked specimens. A detailed analysis of local carbonation ingress around crack faces and in regions remote from cracks then ensues. The results provide the design basis for functionally layered concrete beams that concurrently meet durability performance and lower embodied carbon aspirations.

## The interaction of cracking and carbonation

**Fig. 1:**
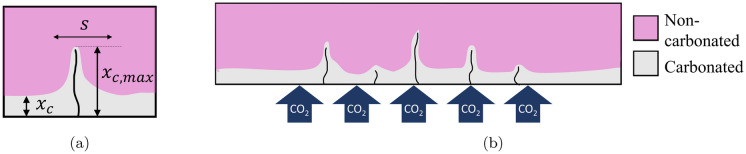
Carbonation behaviour around concrete cracks

In conventional reinforced concrete beams and slabs, flexural cracks typically initiate perpendicular to the soffit at the extreme concrete tensile fibre. Such cracks cross tensile steel reinforcement, thus opening a direct path for the ingress of aggressive substances such as carbon dioxide and chlorides (see Fig. 1). This potentially increases the propensity for steel reinforcement to corrode. As such, BS EN 1992-1-1:2004 [11] (Eurocode 2) enforces a maximum structural crack width to help ensure a minimum durability performance of reinforced concrete structures.

The carbonation depth $x_c$ in an uncracked free face region is generally modelled using Fick’s first law [12]. Accounting for an initial depth of carbonated material $x_0$ at *t*=0 using a non-linear approach [13] then leads to:

1 $$\begin{aligned} x_c=\sqrt{x_0^2+K^2t} \end{aligned}$$

where *K* is the carbonation coefficient. However, flexural cracks can reduce the carbonation resistance of a reinforced structural element since CO$_2$ molecules travel along the crack and penetrate into the concrete. The carbonation front therefore extends deeper into a specimen at crack locations to a maximum penetration depth, $x_{{c,max }}$, as defined in Fig. 1a. The region surrounding the crack in which these effects occur is referred to as the crack influencing zone, with width *s*. As steel reinforcement is vulnerable to carbonation influences, the width of the crack influencing zone at the depth of the reinforcement is of particular interest.

Two-dimensional diffusion models have been proposed e.g. [14, 15] to calculate the influence of cracking on carbonation into concrete perpendicular to a crack direction and along its length. Gil’mutdinov et al. [16] have also developed a semi-empirical model for carbonation depth in the presence of cracks which involves applying adjustment factors to a Fickian diffusion relationship similar to Eq. 1. Studies have suggested that the propagation in the region of the crack depends not only on the crack length but also on the crack width. At very large crack widths, CO$_2$ can diffuse freely and the crack surface may behave equivalently to a free face [17]. With small crack widths, there is more limited progression expected because the crack itself does not permit free transport of CO$_2$. It has been established that greater crack widths lead to greater maximum penetration of carbonation into concrete, and the locations of these maxima are coincident with cracks [18–22].

To quantify the influence of crack width on surrounding carbonation, De Schutter studied a series of mortar specimens with cast-in artificial cracks of fixed length and width, and exposed them to a 10% CO$_2$ environment [23]. A crack influence factor, $\gamma (w,d)$, was defined as the ratio of the carbon diffusion resistance of the artificially cracked specimen *K*(*w*, *d*) relative to that of the uncracked specimen $K(w=0, d=0)$:

2 $$\begin{aligned} \gamma (w,d)=\frac{K(w,d)}{K(w=0, d=0)} \end{aligned}$$

where the crack width is *w* and the crack depth is *d*. Based on the maximum carbonation values, De Schutter found that a crack influence factor of $\gamma >1.8$ was possible for a crack width of 0.5mm and a crack depth of 10 mm.

## Experimental programme

An experimental investigation was undertaken on two-layer functionally layered concrete beams to probe the influence of flexural cracks on the carbonation ingress. The beams were cracked, unloaded and then were reloaded and clamped to maintain target crack widths while exposed to accelerated carbonation conditions. The resulting carbonation fronts were measured and analysed. The experimental design and methodology is detailed in the following.

### Experimental design of layered concrete beams

Reinforced concrete beams with two horizontal concrete layers and a single layer of longitudinal reinforcement were subjected to four-point bending to induce cracks. This design suited the research objectives to create flexural cracks in a controlled manner prior to carbonation exposure. To inform the experimental design, the elastic behaviour up to first cracking was analysed using a transformed section method. In the cracked elastic state, the concrete compression zone was modelled as linear-elastic and a no-tension model was applied. The moment at first yield and ultimate failure moment were calculated using equilibrium, assuming a linear elastic or non-linear stress block for the compression zone respectively. The beams were thus designed to be under-reinforced. The transformed approach and resultant equations for the two-layer beams subjected to bending can be found in [24, 25], and agreed with those derived elsewhere for the general case with any number of layers [26].

### Experimental test parameters

**Fig. 2:**
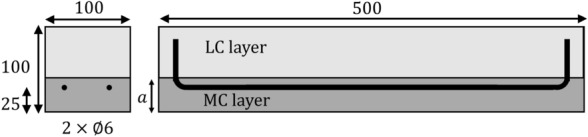
Schematic diagram of beam reinforcement layout and layer configuration, dimensions in mms

A total of 10 reinforced concrete beams with dimensions 100 mm $\times$ 100 mm $\times$ 500 mm were cast, containing up to two horizontal layers of concrete with two different mix compositions. The concrete mixes were identified as ‘MC’ for medium cement content and ‘LC’ for lower cement content. The thickness of the MC layer is denoted as *a*. The reinforcement ratio in all specimens was $\rho =0.57\%$, consisting of two 6 mm diameter bars with a yield strength of 530 MPa. The cover depth was set at 25 mm (Fig. 2).

Layer thicknesses of $a=15$ mm, $a=25$ mm, and $a=40$ mm were selected to represent a range between the likely minimum and maximum thickness of a durability layer. The 15 mm layer was contained within the cover to reinforcement, whereas the 25 mm layer was equal to the concrete cover. The 40 mm layer fully encased the reinforcement. Reference specimens containing just a single concrete mix were also included. Two specimens of each layer thickness were exposed to an accelerated carbonation environment with flexural cracks held open at nominally ‘large’, $w\approx$ 0.3 mm, or ‘small’, $w\approx$ 0.15 mm, widths at the extreme fibre.

The large crack width (LW) was chosen to be consistent with the maximum crack width typically allowed in design codes, e.g.[11], whereas the small width (SW) represented half that value, and is commonly reported as a low crack width in studies elsewhere e.g.[20]. The nomenclature of the resulting experimental test matrix is given in Table 1.

**Table 1:** Test matrix of crack widths and layer thicknesses explored

	Reference	$a=15$ mm	$a=25$ mm	$a=40$ mm	Reference
				
$w\approx 0.15$ mm	LC100.SW	MC15LC85.SW	MC25LC75.SW	MC40LC60.SW	MC100.SW
$w\approx 0.3$ mm	LC100.LW	MC15LC85.LW	MC25LC75.LW	MC40LC60.LW	MC100.LW
Relative cement [%]	75	79	81	85	100

The nomenclature adopted to uniquely identify each of the 10 beams indicates the mix compositions and thickness of each concrete layer in mm, followed by either ‘SW’ or ‘LW’, for small width and large width cracks respectively. For example, MC15LC85.SW denotes the specimen with a 15 mm layer of MC concrete, 85 mm layer of LC concrete and small target crack widths, whereas LC100.LW indicates a specimen containing only a single 100 mm layer of LC concrete, with large target crack widths.

### Material properties

**Fig. 3:**
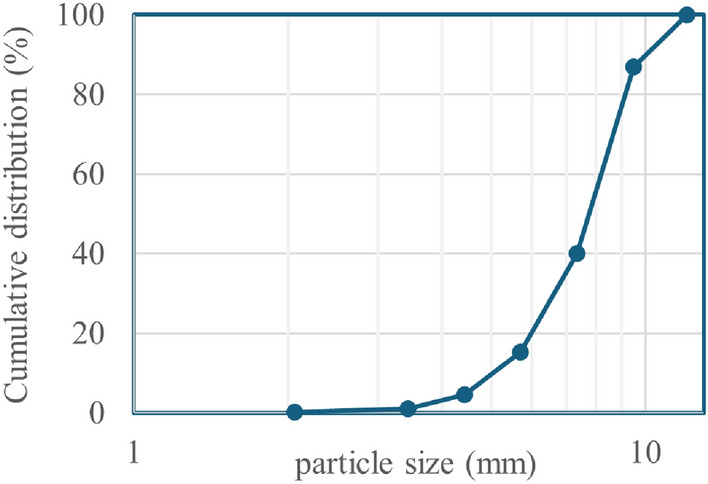
Coarse aggregate grading curve

The concretes contained Portland Limestone Cement CEM II/A-L 32.5R corresponding to a Portland cement with a strength class of 32.5, rapid early strength and between 6-20% limestone fine additions. The LC and MC concretes were designed with water/cement (*w*/*c*) ratios of 0.6 to 0.8 to demonstrate the relative resistance to carbonation in the accelerated study, even though it is unlikely that such high *w*/*c* ratios are used in practice. Previous carbonation testing on similar mixes [24] indicated that the 0.6 *w*/*c* mix had a higher carbonation resistance than that of the 0.8 *w*/*c* concrete. This therefore personifies in a relative sense a protective durability layer. The aggregates consisted of sand (0–4 mm) and gravel (0–12 mm). The particle size distribution within the gravel coarse aggregate was measured using a dynamic image analyser (Sympatec QICPIC). The corresponding grading curve can be found in Fig. 3. The mix designs are given in Table 2. The mix designs were generated using the BRE method of design of normal concrete mixes [27]. In the BRE method, a target mean compressive strength for a given cement strength is used to find the free water to cement ratio. The desired workability depends on the maximum aggregate size, fine and coarse aggregate grading and aggregate type e.g. crushed or uncrushed, which is linked to the fine and coarse aggregate proportions and total aggregate to cement ratio. A small amount of black mortar dye was included in the MC mix for easy visualisation of the layers. The corresponding relative cement percentages for each beam are also included in Table 1 as an indicator of the relative embodied CO$_2$ emissions. This was calculated by dividing the total cement used in a given beam by that of MC100.

**Table 2:** Mix proportions and concrete properties

			‘LC’	‘MC’
Mix composition	CEM II/A-L 32.5 R	[kg/m$^3$]	250	333
	Sand 0–4 mm	[kg/m$^3$]	792	683
	Gravel 0–12 mm	[kg/m$^3$]	1008	1024
	Mortar Dye	[kg/m$^3$]	–	5
	Water	[kg/m$^3$]	200	200
	*w*/*c*	[-]	0.8	0.6
Properties	$f_{cu,28d}$	[MPa]	$\mu =$ 25.0, $\sigma =$ 0.3	$\mu =$ 37.2, $\sigma =$ 2.7
	Slump	[mm]	23	24

### Layered casting

The specimens were cast using a wet-on-wet process [28], whereby each concrete layer was cast in the fresh state with minimal pour-delay, to mitigate the formation of a cold-joint between layers. The slump of the fresh state mixes were measured in accordance with BS EN 12350-2 [29]. Reference 100 $\times$ 100 $\times$ 100 mm cube specimens, subsequently carbonated in a non-loaded state or used as hardened state compression control specimens, were also cast.

The specimens for carbonation exposure were cured in water for 7 days and then transferred to a standard laboratory environment. Compression control test specimens were water cured for 28 days.

### Pre-cracking, clamping and carbonation exposure

**Fig. 4:**
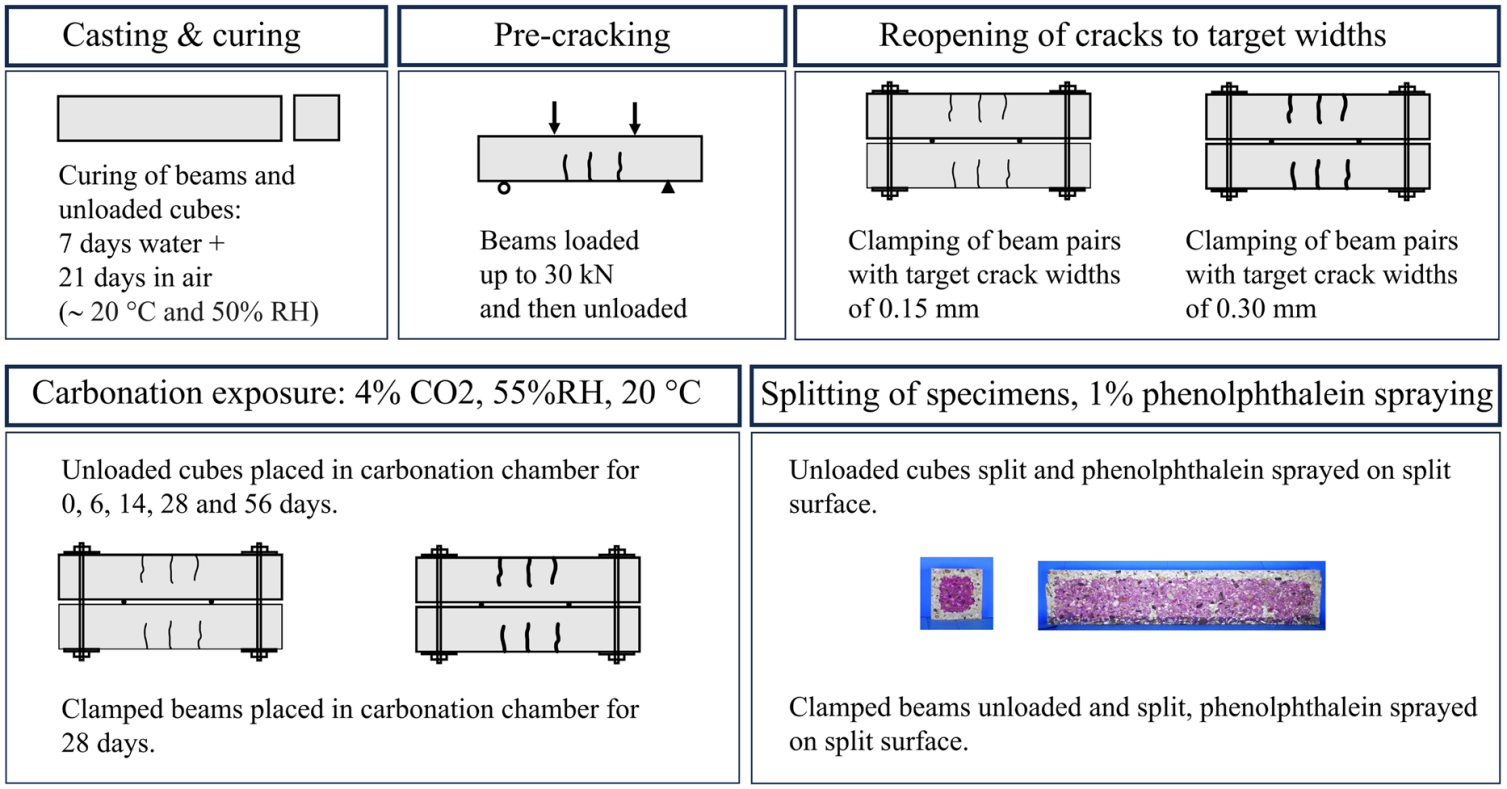
Experimental methodology

An overview of the experimental methodology is shown in Fig. 4. Further details of the pre-cracking, clamping and exposure conditions can be found in the following.

#### Pre-cracking

The beam specimens were pre-cracked at an age of 28 days. The compressive cube strengths, $f_{cu,28d}$, also measured at age 28 days are given in Table 2 where the mean $\mu$ and the standard deviation $\sigma$ as derived from three cube tests are shown.

**Fig. 5:**
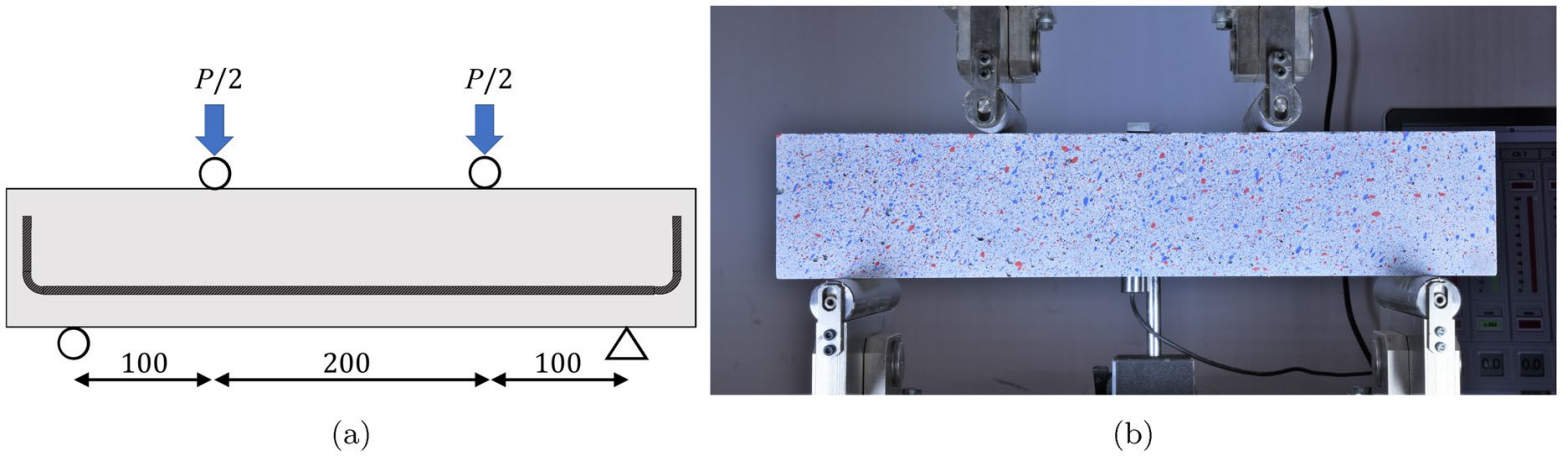
Pre-cracking configuration (**a**) schematic with dimensions in mms (**b**) specimen in test frame

A four-point bend test method (BS EN 12390-5 [30]) was used to induce flexural cracks in all specimens. The distance between the beam supports was 400 mm, with the shear spans equal to 100 mm and flexural span between loading points equal to 200 mm. The loading configuration is shown in Fig. 5. The mid-span deflection was measured using a central span transducer, and the loading rate was displacement controlled up to a fixed load of 30 kN, to generate cracks. The load was defined such that the beams would be within the cracked elastic regime and was equal in all beams, regardless of whether they would be re-opened to small (SW) or large (LW) crack widths during carbonation exposure.

During the cracking stage, three or four flexural cracks were generated in the central span in all ten specimens. The presence of the durability layer generally increased the load at first cracking, the uncracked stiffness and the cracked elastic stiffness relative to the LC beam. Hence, the inclusion of the fresh-on-fresh cast durability layer did not have a negative impact on these properties.

#### Clamping

**Fig. 6:**
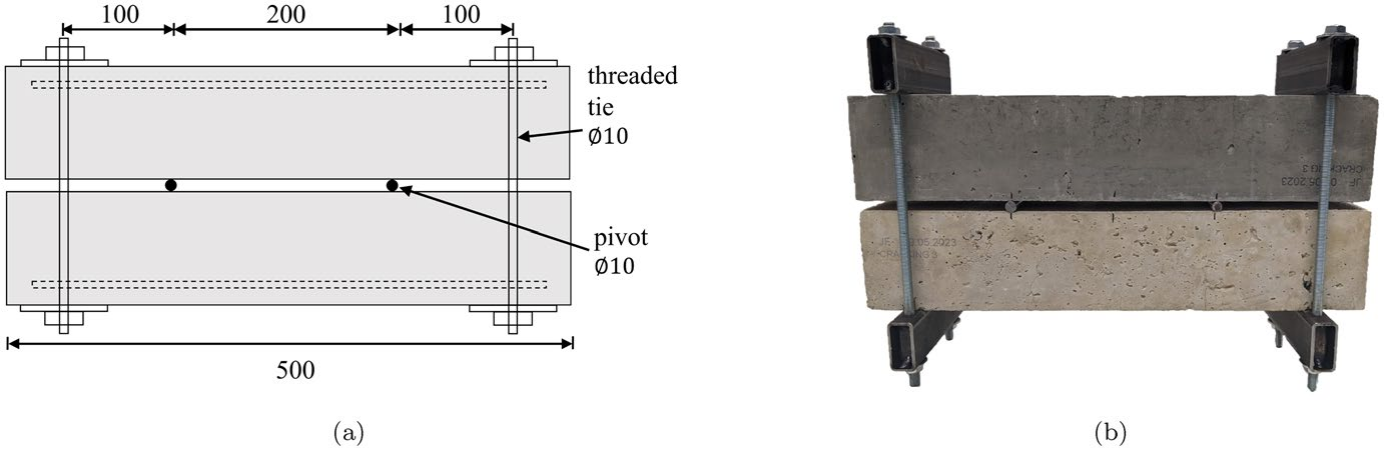
Crack generation for carbonation exposure (**a**) clamped specimen layout (dimensions in mms) (**b**) photo of clamped specimens

**Fig. 7:**
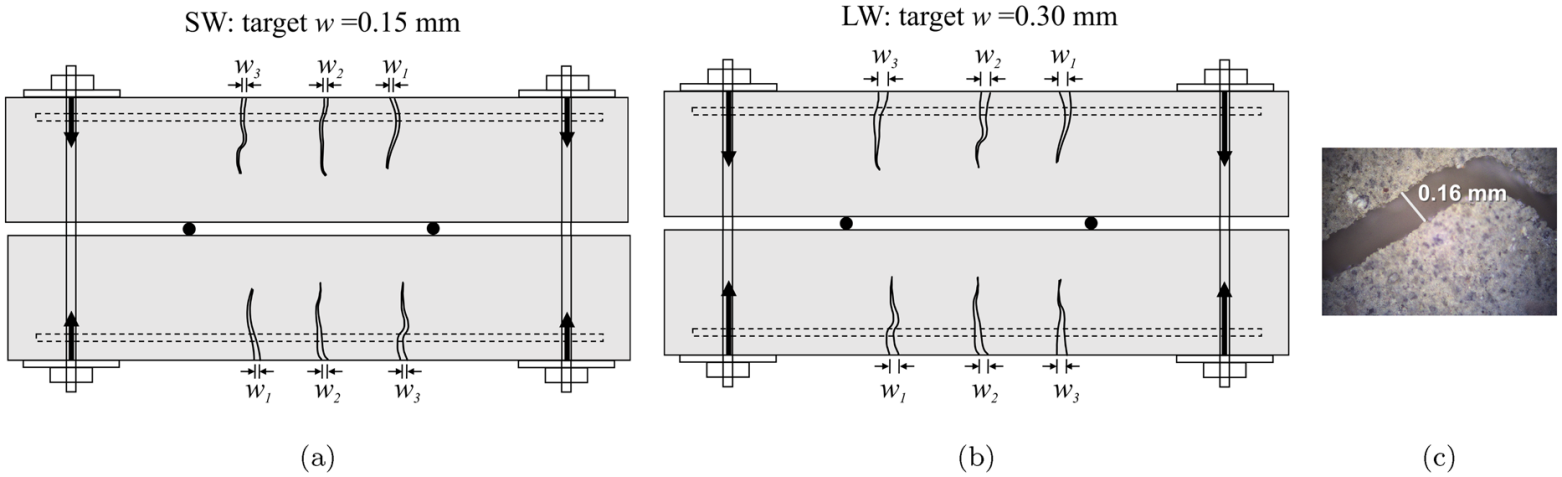
Schematic diagrams of loaded beams with (**a**) target average crack widths of 0.15 mm and (**b**) target average crack widths of 0.30 mm (**c**) crack width measurement

The beams were then unloaded and removed from the test frame. They were subsequently clamped in pairs using the configuration shown in Fig. 6. The existing cracks in the tensile face were opened in both specimens of a pair, to as near as possible to 0.15 mm (SW) or 0.3 mm (LW) crack widths, as shown in Fig. 7 (a) or (b) respectively. The crack width was measured with a portable microscope with LED illumination and a 40 $\times$ objective lens. Images of the microscope measurement of the crack width were captured with a digital camera with a 3.1 MP resolution mounted on the microscope. In all cases the crack widths on the tensile faces were measured and the maximum width for a given crack was taken as the measured crack width. However, as shown in the image in Fig. 7c, even across the width of the beam, the crack width varies and the crack edges are jagged. The width also varies with depth. It is therefore of note that the tensile face crack width may not be representative of the internal cracking associated with the extracted carbonation profiles and should be considered as an indicative measure. Prior to carbonation exposure, the width of each crack at its mouth was recorded along with the locations of each of the cracks, as compiled in Table 3. In Table 3 the distance from the beam end to crack number *i* is indicated as $d_i$ and the corresponding crack mouth width denoted as $w_i$. One challenge with the clamping method is that the width of an individual crack cannot be controlled without affecting the widths of the other cracks in the beam. Therefore, a balance was sought to obtain the target crack widths in an average sense. However, in some cases this led to a disparity between the crack widths. For example, MC15LC85.LW crack 1 had a width of 0.39 mm whereas crack 3 had a width of 0.18 mm, and MC40LC60.LW crack 1 had a width of 0.44 mm while that of crack 2 was only 0.24 mm. Cases where an individual crack width differs from the beam average by more than 20% are noted in bold text and will be discussed further in later sections.

**Table 3:** Crack locations and widths prior to carbonation exposure

Specimen	Crack 1		Crack 2		Crack 3		Crack 4		Average
	$d_1$	$w_1$	$d_2$	$w_2$	$d_3$	$w_3$	$d_4$	$w_4$	$w_{ave}$
	(mm)	(mm)	(mm)	(mm)	(mm)	(mm)	(mm)	(mm)	(mm)
LC100.SW	165	0.18	265	0.16	365	0.18	n/a	n/a	0.17
LC100.LW	155	0.36	225	0.28	335	0.34	n/a	n/a	0.33
MC15LC85.SW	140	0.15	235	0.14	350	0.16	n/a	n/a	0.15
MC15LC85.LW	195	**0.39**	310	0.37	405	**0.18**	n/a	n/a	0.31
MC25LC75.SW	145	0.16	275	0.15	365	0.15	n/a	n/a	0.15
MC25LC75.LW	165	0.31	245	0.28	360	0.30	n/a	n/a	0.30
MC40LC60.SW	170	0.15	210	0.12	285	**0.10**	365	0.15	0.13
MC40LC60.LW	130	**0.44**	220	**0.24**	290	0.30	375	0.39	0.34
MC100.SW	120	0.14	180	0.10	240	0.12	320	0.13	0.12
MC100.LW	170	0.24	275	0.30	365	0.28	n/a	n/a	0.27

#### Carbonation exposure

The clamped specimens were placed in an accelerated carbonation chamber at 4% CO$_2$, 55% relative humidity and 20°C for 28 days. The CO$_2$ concentration was chosen to balance the time for measurable carbonation ingress and need for representative reactions. Companion cubes of the same concrete mixes were also placed into the chamber, to measure the carbonation progression in the non-loaded state alongside the loaded state beams. Due to size constraints of the chamber, beams of LC100, MC15LC85, MC25LC75 and MC100 were exposed in the first instance, and beams of MC40LC60 were exposed in a subsequent period.

Upon removal from the accelerated carbonation environment, the beam specimens were split longitudinally along the central axis using a line load applied by a hydraulic loading jack to reveal an internal surface. The companion cubes were split using a split cylinder apparatus. The split surfaces were sprayed with 1% phenolphthalein in ethanol solution, which turns magenta in contact with non-carbonated concrete, to demarcate the carbonated material. The phenolphthalein test is a simple method of measuring the extent of carbonation based on pH change in carbonated concrete. This approach assumes a sharp front between carbonated and non-carbonated material and does not capture the nuances of the partially carbonated zone and the degree of carbonation. However, it is adequate for indication that carbonation has penetrated to a lesser or greater extent in compared samples.

### Digital image extraction of carbonation profiles

**Fig. 8:**
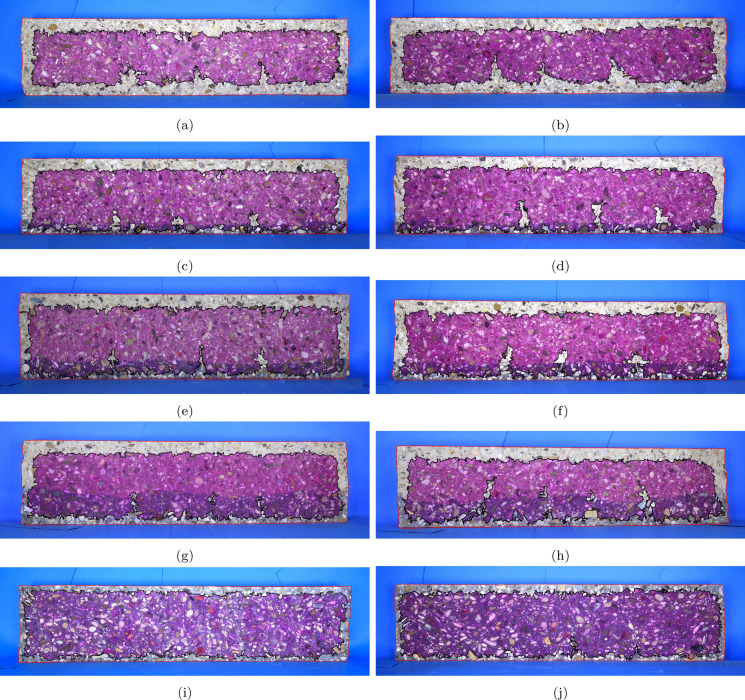
Photographs of carbonation fronts on split beam specimens from top to bottom increasing thickness of MC layer, small crack widths on the left, large crack widths on the right (**a**) LC100.SW (**b**) LC100.LW (**c**) MC15LC85.SW (**d**) MC15LC85.LW (**e**) MC25LC75.SW (**f**) MC25LC75.LW (**g**) MC40LC60.SW (**h**) MC40LC60.LW (**i**) MC100.SW (**j**) MC100.LW

Figure 8 shows photographs of the carbonation fronts observed after spraying the split surfaces of the pre-cracked beams with phenolphthalein indicator solution. The carbonation fronts are irregular, with ingress from all four sides. In addition, the local profiles around the crack faces are complex and difficult to manually trace. Hence, image processing techniques were used to assist with the interpretation by extracting the carbonation fronts from digital photographs of the split faces of the specimens. Using the Matlab Image Processing Toolbox, thresholds were applied in a colour space (in this instance RGB) to isolate the carbonated regions and the edge of specimens in the photographs. For carbonation identification, a single colour threshold was applied to differentiate between grey and pink regions. This was then used to define the boundary of what is referred to as the carbonated region for the remainder of the paper albeit noting the limitations previously mentioned regarding phenolphthalein testing and the assumption of a sharp front. The dimensions in pixels of the subsequent masks were converted to millimetres using reference dimensions such that the depths of the carbonation fronts could be determined. For further details of the approach for detecting carbonation fronts using image processing tools, please see [31].

**Fig. 9:**
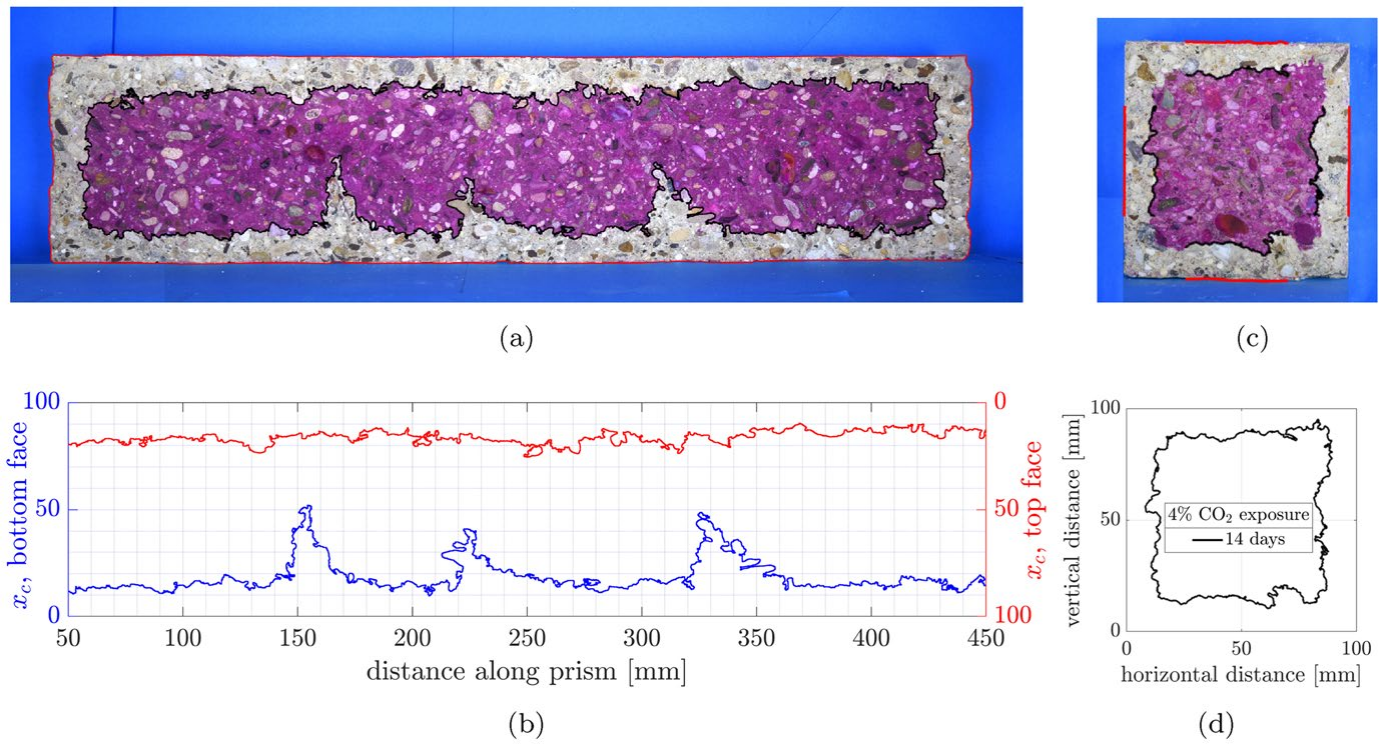
Image processing of carbonation fronts: (**a**) LC100.LW phenolphthalein indication (**b**) LC100.LW image processed carbonation profiles (**c**) LC100 companion cube phenolphthalein indication with 50 mm regions from which carbonation coefficients back-calculated (**d**) LC100 cube image processed carbonation profile

The carbonation depths were thus extracted from the images in Fig. 8. An example for LC100.LW is shown in Fig. 9a and b. The carbonation fronts from the tensile (bottom) and compressive (top) faces are shown as blue and red lines, respectively. These provide deeper insight into not only the cracked regions but also the uncracked compression and tensile regions away from the cracks.

### Carbonation profiles in non-loaded cube specimens

Use of image processing techniques also enabled more comprehensive data to be extracted from the non-loaded companion carbonated cube specimens. In conventional practice for the carbonation exposure and testing of concrete cubes, for example BS EN 12390-12:2020 [32], three of the cube faces are sealed to promote one dimensional diffusion. However, here the cubes were not sealed, such that carbonation into four exposed faces could be measured, as shown in Fig. 9c and d. This was chosen to provide more extensive data from a single test. Two-dimensional diffusion effects around the corner regions are evident, but their effects were ameliorated by considering only the average carbonation depth progression in the central 50 mm of the top, bottom and side faces, thus removing the influence of corner effects (the 50 mm lengths are noted in Fig. 9c).

**Fig. 10:**
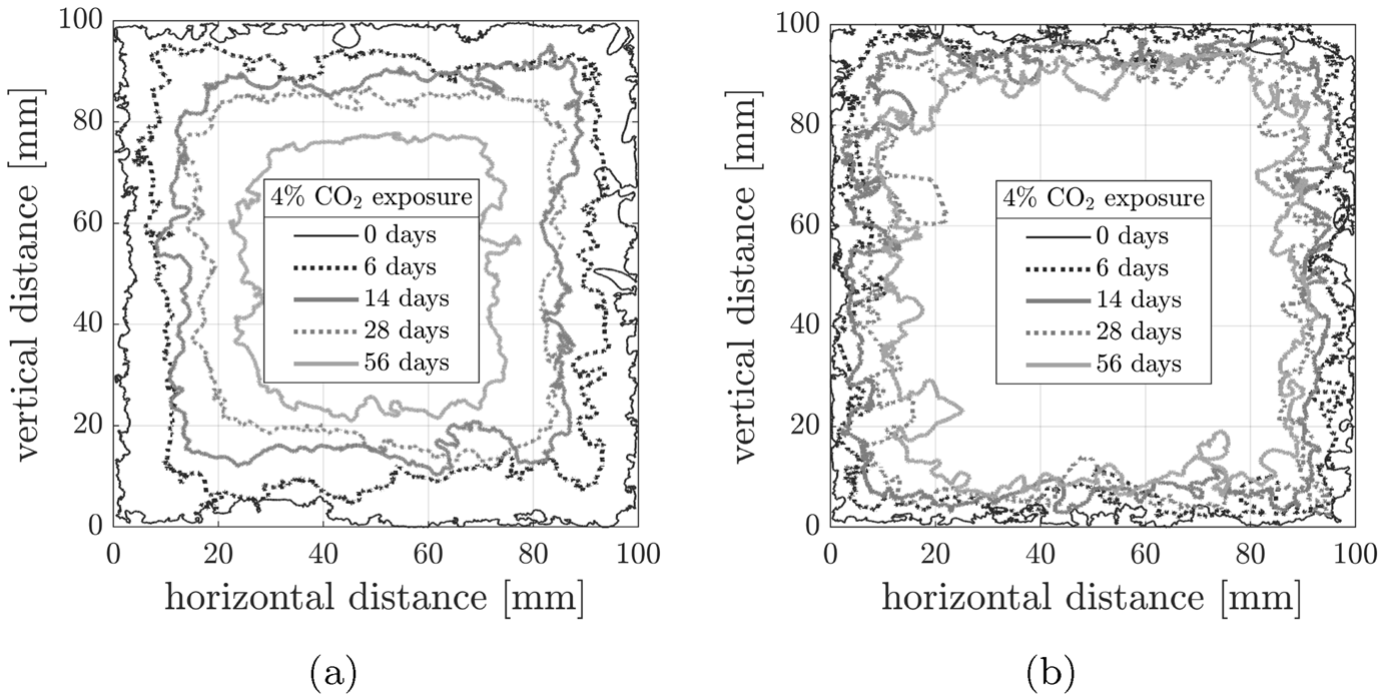
Image processing of non-loaded cube carbonation fronts: (**a**) LC100 cube (**b**) MC100 cube

**Fig. 11:**
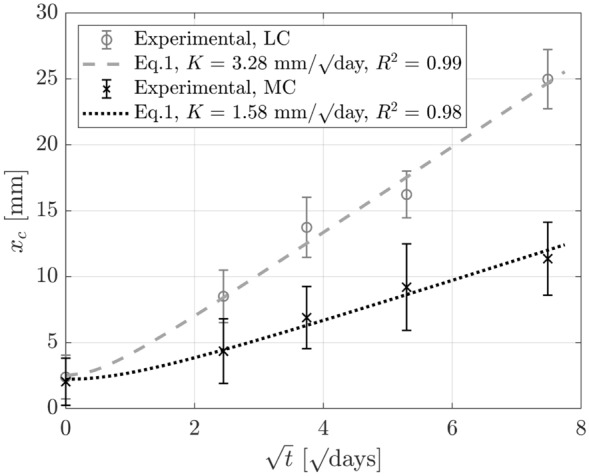
Carbonation depth progression in non-loaded specimens. Error bars indicate one standard deviation in the carbonation depth measured, obtained using image processing tools

The carbonation fronts for the LC100 and MC100 companion non-loaded cube specimens of each mix after 0, 6, 14, 28 and 56 days of accelerated carbonation are given in Fig. 10. Image processing enabled the independent calculation of the carbonation depths in the top, bottom and side-cast faces. The values determined for the different faces were similar, despite previous studies from the authors and elsewhere indicating differences in behaviour due to casting orientation [24, 33, 34]. Therefore, the baseline carbonation depth in the non-loaded state is herein taken as the average of all 4 sides. The carbonation depths of the non-loaded cubes were then plotted against the square root of exposure time in Fig. 11. The estimated carbonation coefficients obtained by fitting Eq. 1 gave $R^2$ values close to 1 suggesting good agreement. With a carbonation coefficient of 1.58 mm/day$^{1/2}$, the carbonation rate in the higher cement mix, MC100, was consistently lower than in the lower cement mix, LC100, which has a coefficient of 3.28 mm/day$^{1/2}$.

## Carbonation profiles in cracked beams as derived from digital images

The carbonation depths in the beam specimens were extracted from the digital carbonation fronts. The compression face, tensile regions remote from cracks and tensile regions adjacent to cracks were identified as three distinct regions of interest and were quantified using the following approaches.

### Compression face carbonation

**Fig. 12:**
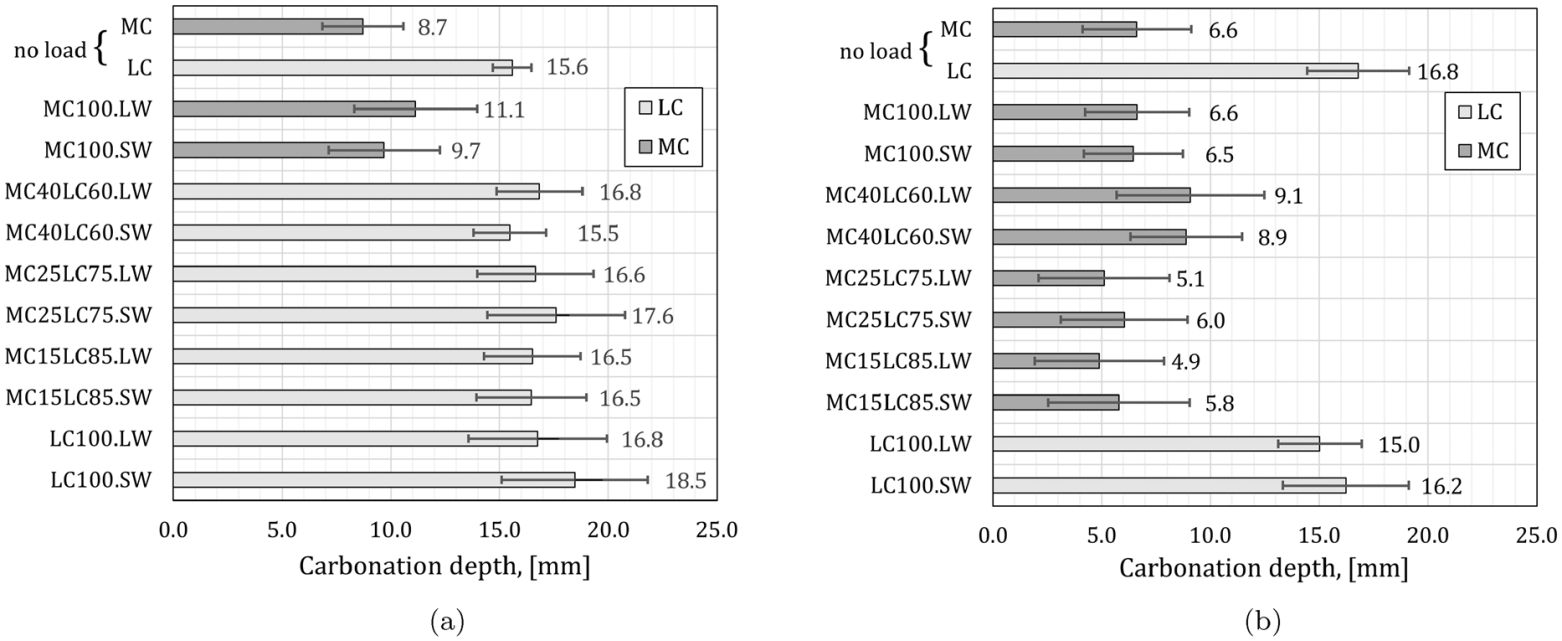
Carbonation depths after 28 days of 4% CO$_2$ exposure in non-loaded cube specimens and in beam regions remote from cracks for the (**a**) top-cast compression and (**b**) bottom-cast tensile faces. The error bars indicate one standard deviation in measurements

The compressive face of the beam was uncracked and so the average carbonation depth and standard deviation were derived from the readings along the entire top face taken after 28 days of exposure. The results are presented in Fig. 12a. The carbonation depths from the top (trowelled) faces of the non-loaded companion cube specimens are included for comparison and denoted as ‘no load’ MC or LC results.

### Tensile face carbonation

The carbonation profiles on the beam tensile faces were highly non-uniform so the behaviour was separated into two regions: regions remote from cracks, and regions adjacent to cracks. The bottom face regions remote from cracks used the average carbonation depth in regions greater than 25 mm away from the cracks whereas in the regions adjacent to cracks 25 mm widths either side of each crack were considered.

#### Regions remote from cracks

The average carbonation depths along the beam tensile face remote from cracks, are presented in Fig. 12b. The carbonation depths from the bottom (cast) faces of the non-loaded companion cube specimens are included as ‘no load’ MC or LC results.

#### Regions adjacent to cracks

**Fig. 13:**
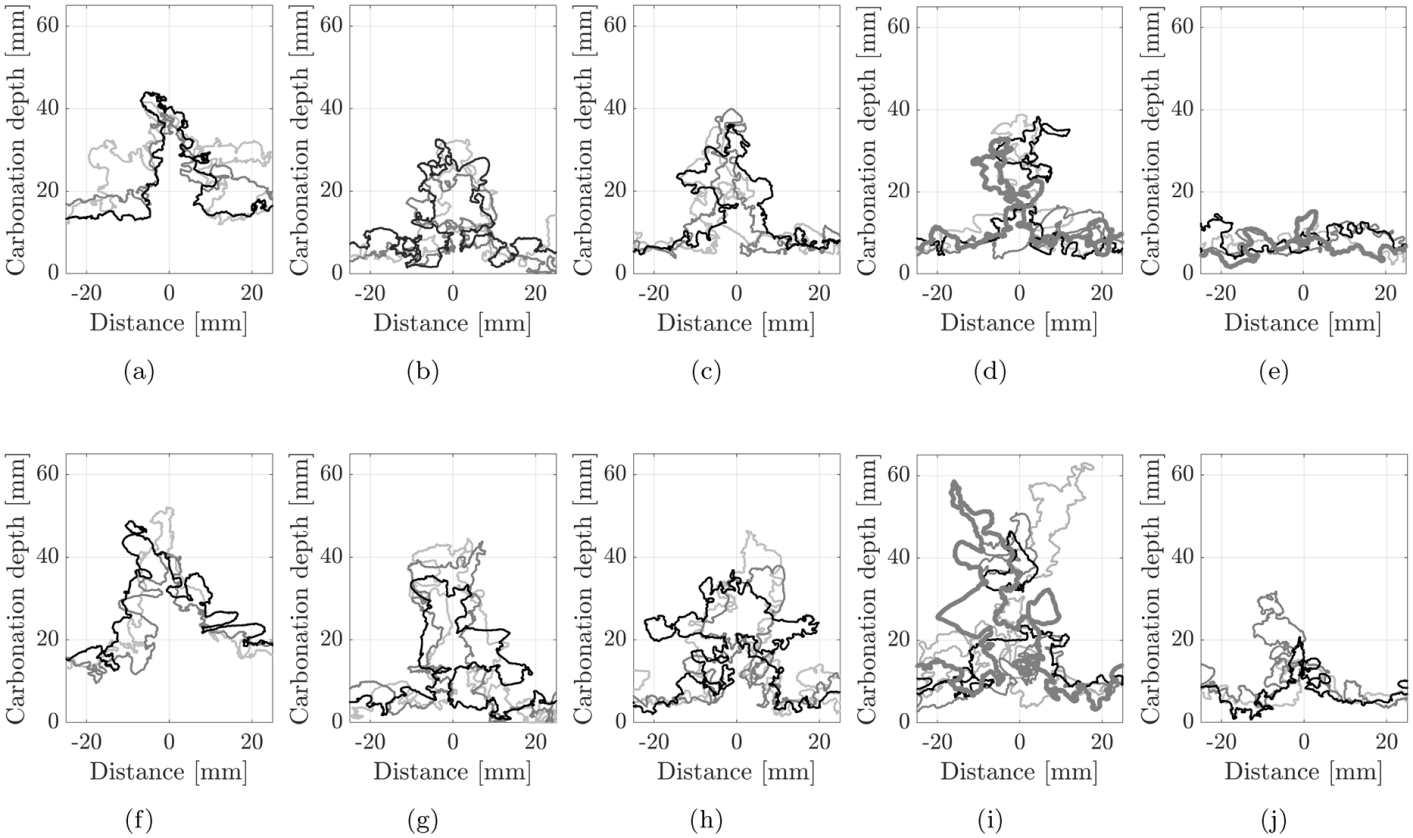
Crack carbonation details (**a**) LC100.SW (**b**) MC15LC85.SW (**c**) MC25LC75.SW (**d**) MC40LC60.SW (**e**) MC100.SW (**f**) LC100.LW (**g**) MC15LC85.LW (**h**) MC25LC75.LW (**i**) MC40LC60.LW (**j**) MC100.LW

**Fig. 14:**
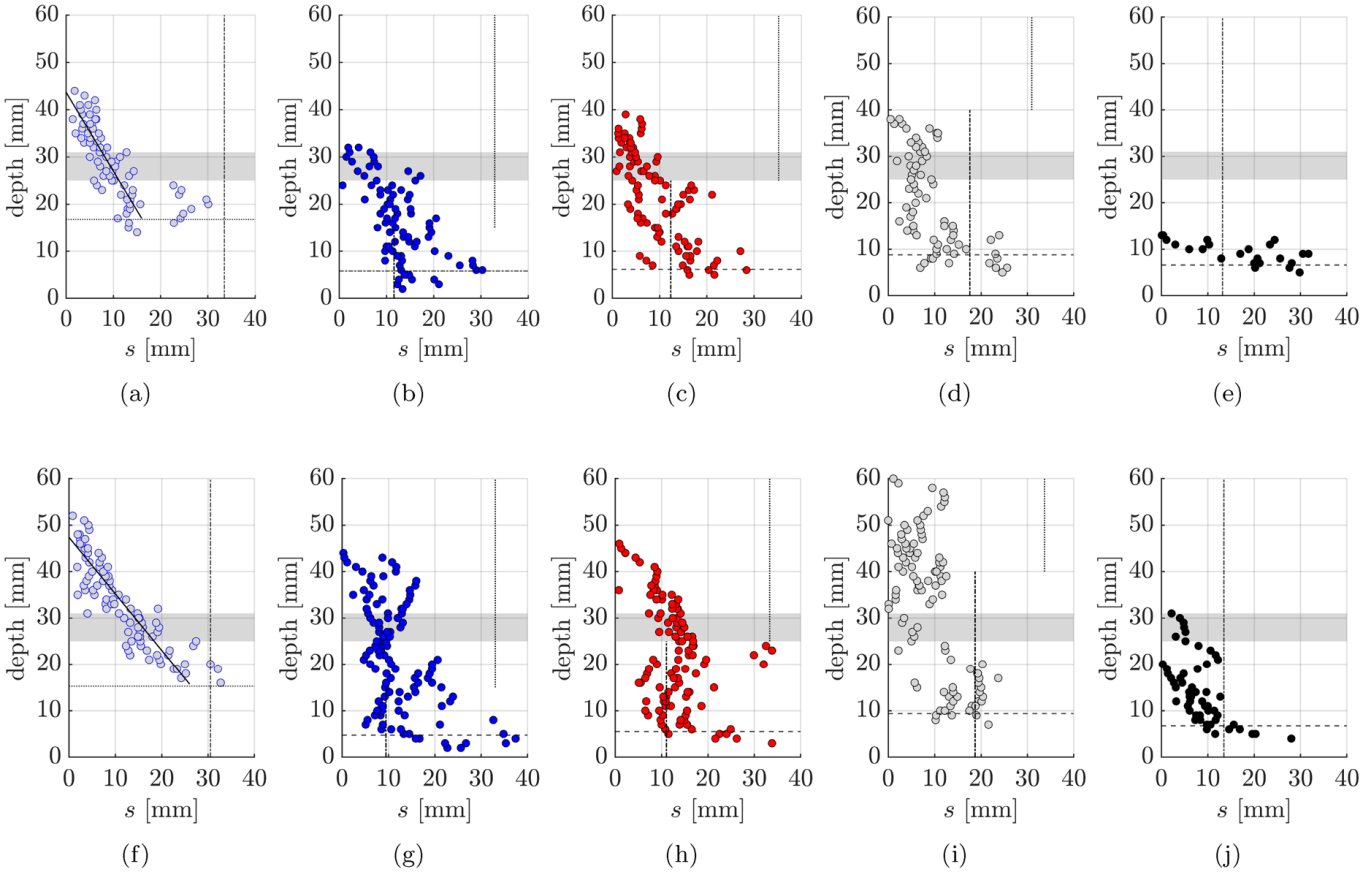
Discretised crack influencing zone (**a**) LC100.SW (**b**) MC15LC85.SW (**c**) MC25LC75.SW (**d**) MC40LC60.SW (**e**) MC100.SW (**f**) LC100.LW (**g**) MC15LC85.LW (**h**) MC25LC75.LW (**i**) MC40LC60.LW (**j**) MC100.LW

The digital image results for the region ± 25 mm from the crack mouth location were plotted for all the cracks in a given beam and are presented for the small crack width (SW) and large crack width (LW) specimens in Fig. 13. The profile of the width of the carbonation region surrounding the crack, *s*, was further interrogated by discretising the results. At depth increments of 1 mm the width of the region of influence for a given crack face was extracted from the image processing plots. Hence if the width of carbonation extended from −5 mm to $+$12 mm from the crack mouth location, the total width *s* of 17 mm would be plotted in Fig. 14 against the depth in the vertical direction. This approximation acted to accommodate the different crack shapes as well as to somewhat smooth the inherent irregularity of the profiles due to factors such as the variability, tortuosity of the crack and the influence of individual aggregates. In Fig. 14, the horizontal lines indicate the mean $x_c$ free face carbonation depths (using the bottom face remote from cracks values presented in Fig. 12). The vertical lines show 2$x_c$ which would indicate the total penetration width if both sides of the crack behaved in an equivalent manner to a free face (using the values in Fig. 12 for the appropriate concrete layer). The shaded region indicates the location and extent of the embedded steel reinforcement.

**Fig. 15:**
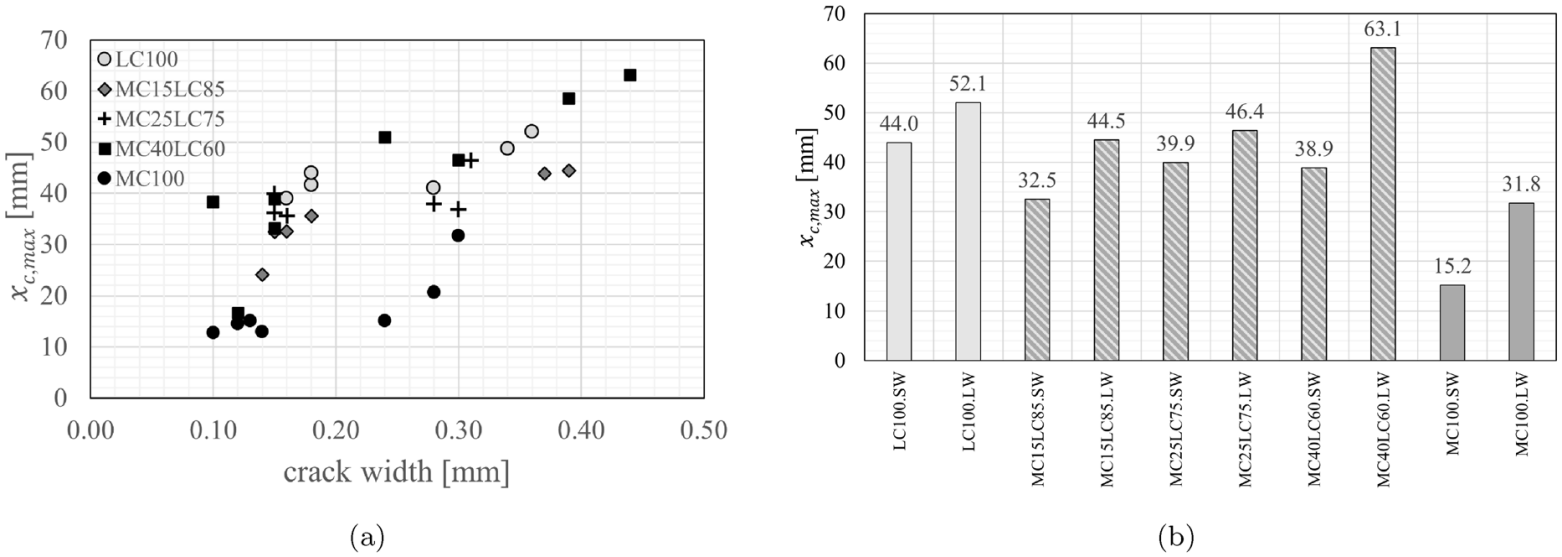
Maximum penetration depths (**a**) *x*_*c,max*_ vs *w* for all cracks and (**b**) maximum carbonation depths *x*_*c,max*_ in cracked beams

For each crack location the maximum carbonation depth, $x_{{c,{ {max}}}}$, is obtained from the profile of the carbonation ingress. In Fig. 15a, the crack width *w* at the crack mouth of each crack measured prior to carbonation was plotted against the maximum carbonation depth $x_{{c,{ {max}}}}$ at that crack. The maximum $x_{{c,{ {max}}}}$ values across all the cracks in a given beam designation are then summarised in Fig. 15b.

## Discussion of carbonation in cracked functionally layered beams

General trends relating to the carbonation behaviour of cracked functionally layered beams can be noted based on Figs. 8 and 13. Broadly speaking, within the depth of the outer MC durability tensile layer, the free face carbonation in the layered beams has not penetrated to the same extent as the LC100 sample. Thus the width of carbonation penetration adjacent to the crack mouth is localised around the crack face. The carbonation front shapes in the regions adjacent to cracks indicate that two-dimensional diffusion has occurred, whereby CO$_2$ has diffused in from the free face as well as through the crack itself. Since the top layer concrete had a lower carbonation resistance than the durability layer, the width of the influencing zone is in some cases equal to or greater in the top layer than in the durability layer even though the crack widths closer to the neutral axis would be expected to be smaller.

In the following, the carbonation characteristics derived from the image processing analyses are investigated to discuss the carbonation progression in the functionally layered beams in regions remote from, and adjacent to, cracks. The implications for the design of functional durability layers are also probed.

### Compressive regions and tensile regions remote from cracks

The loaded beam compressive faces, and tensile regions away from the cracks were compared in the first instance. The results in Fig. 12a show that the average carbonation depths, $x_c$, for the compression face of the layered specimens (between 15.5 mm and 17.6 mm) are consistent with the non-loaded LC cube results (15.6 mm) and LC100 loaded beams compressive faces (16.8 mm and 18.5 mm). The layered specimen tensile face carbonation depths (between 4.9 mm and 9.1 mm) shown in Fig. 12b are more similar to the mix MC non-loaded cube (average depth of 6.6 mm) and MC100 loaded beam results (6.5 mm and 6.6 mm). This suggests that the protective durability layer was effective in reducing free face carbonation ingress in regions remote from cracks. It is noted that the MC40LC60 specimens showed a slightly higher carbonation depth in the bottom face (8.9 mm and 9.1 mm) than the other bottom faces of the MC mix, though still within the error bounds shown. These specimens had one more crack (four) than the majority of other specimens, which had three. Therefore, the tensile ‘remote from cracks’ carbonation depth values are obtained from a smaller number of measurements and are more sensitive to the presence of large aggregates.

RILEM TC 281-CCC [33] conducted round robin testing on the effect of uniaxial stress ratios of 30% and 60% on the carbonation behaviour of different concretes under CO$_2$ concentrations of either 2% or 20%. It was found that carbonation rates in Portland Cement specimens subjected to uniaxial compressive loads could be higher or lower compared to non-loaded specimens, depending on the load level. Uniaxial tensile stress experiments displayed increased carbonation rates [33]. In the results reported here, the applied compressive and tensile flexural stresses vary through the depth of the beam so the applied stress and carbonation progression are difficult to decouple. Nevertheless, the flexural stresses did not appear to significantly affect the carbonation ingress since the carbonation depths in the non-loaded specimens did not differ significantly from those in the loaded specimens for the same casting face.

### Tensile regions adjacent to cracks

Figure 15 suggests a positive correlation between the crack width and the maximum carbonation depth $x_{{c,{{max}}}}$ for the single mix and layered specimens. As expected, the LC100 specimen shows higher maximum carbonation depths over the range of widths tested than the MC100 specimen, due to lower material carbonation resistance. This observation is consistent with findings of Wang et al. [35, 36] who found that the concrete composition influenced the carbonation ingress under loaded conditions. Carević and Ignjatović investigations [18, 19] also suggest that larger crack widths lead to greater maximum CO$_2$ penetration. In those studies, sustained cracks were induced in reinforced concrete specimens at an age of 90 days and the beams were exposed to accelerated carbonation conditions for a similar period (28 days) to that used here but at a lower CO$_2$ concentration of 2% and a higher RH of 65 ± 5%. The concrete included 275 kg/m$^3$ of CEM II/A-M (S-L) 42.5R and 175 kg/m$^3$ water. For a crack width of 0.15 mm, the measured $x_{{c,{{max}}}}$ was around 30 mm whereas for a crack width of 0.5 mm $x_{{c,{{max}}}}$ was as high as 50 mm [18, 19]. Since the carbonation penetrated through the crack into the top layer of the LC concrete for all the layered specimens, regardless of the layer thickness, the relationship between the maximum carbonation depth and crack width in the layered specimens was generally in line with the observed results for LC100. Carbonation present in the top layer of the layered beams is presumed to have been caused by crack opening facilitating direct access of the CO$_2$ molecules to the material at this depth, which can then diffuse into the concrete directly. The maximum $x_{{c,{{max}}}}$ was greater in specimens with large crack widths (LW) than small crack widths (SW) within each beam pair (see Fig. 15a). With the exception of MC40LC60.LW, for a given crack width (SW or LW), the layered $x_{{c,{{max}}}}$ values are lower than that of the analogous LC100 mix but higher than that of the analogous MC100 mix. The $x_{{c,{{max}}}}$ values for the SW 15 mm, 25 mm and 40 mm layers were similar (between 32.5 mm and 39.9 mm), as were the LW 15 and 25 mm layers (44.5 and 46.4 mm), suggesting that the layer thickness did not cause a significant difference to the maximum depth of penetration. Further experimental data would be required to confirm the statistical significance of these findings. As can be seen in Table 3 and Fig. 15a, MC40LC60.LW cracks 1 ($w_1$=0.44 mm) and 4 ($w_4$=0.39 mm) had widths in excess of the target width and this potentially led to the greater penetration at these cracks. Theoretically, the cracked elastic no-tension neutral axis should be at a depth of around 80 mm. Yet, the $x_{{c,{{max}}}}$ values are less than this. This suggests that autogenous healing or limited carbonation through crack faces with very small crack widths could play a role. Establishing such thresholds would be the subject of further work.

The width of the crack influencing zone, *s*, provides further insight. The results in Fig. 14 show that in the LC100 beams the lower regions of the tensile face have completely carbonated. This can be contrasted with the layered beams where the outer MC layer leads to more localised penetration at the crack faces. In the LC100.SW beams, the width of penetration at a depth of *y* $\approx$ 20 mm is influenced by carbonation through both the free face and crack face and reaches a value of *s* $\approx$ 30 mm which is similar to what would be associated with free face ingress. The widths of the influence zone at an equivalent depth (20 mm) in the SW layered beams are smaller than LC100.SW. At depths above the steel e.g. > 31 mm, the widths of the influence zones in the layered beams are generally close to that of the LC100 beams. There were no signs of carbonation ingress in MC100.SW above a depth of around 12 mm. The crack influence zones in the LW layered beams differ somewhat across the three (or four) cracks. For example, one of the cracks in MC15LC85 widens at a depth of around 22 mm and the relatively wide zone of influence may be indicative of local cracking at the steel level. Bulbing of the carbonation profile at one of the cracks in MC25LC75.LW occurred at a level of around 20-25 mm which might suggest either local cracking or preferential carbonation penetration along the layer interface. The possibility of carbonation progression along a concrete layer interface would be the subject of further work. In MC40LC60 a zone of ostensibly uncarbonated material is bounded by carbonated material above and below. Through the MC layers, the widths of influence are generally greater than that of the MC100.LW beam. Moreover, the greater localisation around the crack due to the MC layer, and the similarity with LC100.LW at depths above the steel are consistent with the SW observations.

For the LC100 data in Fig. 14, a linear regression fit to the carbonation width data was carried out for $s<$ 20 mm (to exclude the cluster of points with $s>$ 20 mm that appeared to be associated with two-dimensional diffusion) for the SW case and for $y>$ 15 and $s<$ 30 mm for the LW case. The led to $R^2$ values > 0.70 and > 0.75 for LC100.SW and LC100.LW respectively suggesting a correlation between the width of the influence zone and crack depth. The scatter in the layered beams and smaller number of data points within a given layer were not conducive to an accurate linear fit. The smaller zones of penetration in the MC100 beams meant that variability in the carbonation profile due to local aggregates, measurement inaccuracies etc. played a greater absolute role. Another factor, however, is that within a given beam the crack widths differed.

**Fig. 16:**
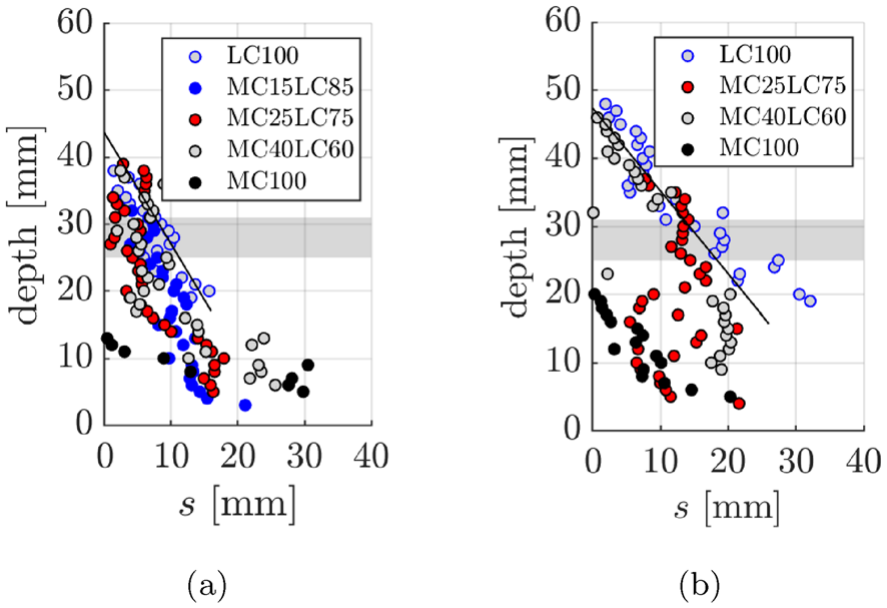
Crack influencing zone for cracks with selected widths of (**a**) 0.14, 0.15 or 0.16 mm (SW beams) (**b**) 0.28 or 0.30 mm (LW beams)

To investigate this further, selected cracks (see Table 3) with widths between 0.14 mm and 0.16 mm: namely crack 2 in LC100.SW and MC25LC75.SW and crack 1 in MC15LC85.SW, MC40LC60.SW and MC100.SW; and selected cracks between 0.28 mm and 0.30 mm including crack 2 in LC100.LW and MC25LC75.LW and crack 3 in MC40LC60.LW and MC100.LW, were superposed in Fig. 16. There was not a MC15LC85.LW crack in the 0.28 mm to 0.30 mm range so it was not plotted. The LC100.SW and LC100.LW linear regression fits from Fig. 14 were also added for comparison purposes. The results provide further evidence that the behaviour of LC100 and MC100 generally bound the zones of influence of the layered beams and that the layered beams have similar carbonation profiles in the LC layer to that of the LC100 concrete above the steel. But in MC40LC60, the embedment of the reinforcement steel in the MC layer has led to a smaller penetration through the steel region.

**Fig. 17:**
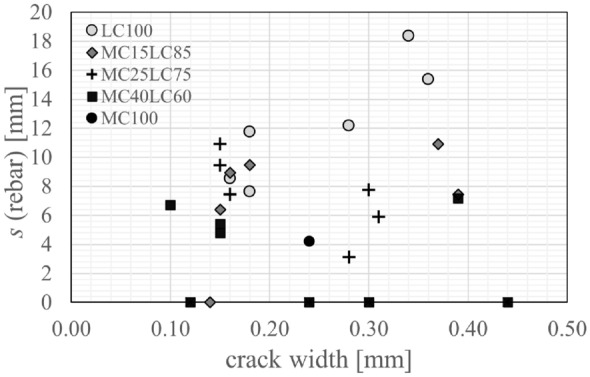
Width of crack influencing zone at level of reinforcement at depth of 28 mm

The width of the carbonation influencing zone at the level of the reinforcement steel (28 mm) is of particular interest for durability performance and so is shown in Fig. 17. The widest width of influence was in the LC100.LW specimen and was measured to be up to 18.4 mm. In contrast, with the exception of one crack which had a penetration width of 4.2 mm, no carbonation was measured at the level of the reinforcement in the MC100 crack locations. For the smaller crack widths, the width of the crack influence zone at the level of the steel for MC15LC85.SW and MC25LC75.SW were broadly similar to that of LC100.SW. However for the larger target crack widths, the functional layering reduced the width of the influence zone relative to LC100. The embedment of the steel in the MC layer, as was the case in MC40LC60, had a positive effect. Across all the measured MC40LC60 cracks, the maximum width of influence was 7.2 mm. However, it is of note that at one of the MC40LC60.SW cracks and three of the MC40LC60.LW cracks, carbonation was observed above and below the steel but not at the level of steel. These cases have been plotted as *s*=0 in Fig. 17. Hence even when carbonation has penetrated into the LC mix, the steel in the MC layer bridging the crack is protected to some extent.

Uncertainty surrounding the measured value of crack width must be considered when interpreting Fig. 15, 16 and 17. The crack width was measured at a single location at the extreme tensile fibre, and this value taken as representative. However, as with all real cracks and due to the nature of concrete with coarse aggregate, the width can vary not only along the height of the crack but also across the width of the beam. As such the crack may have been smaller or greater at the location of the split face. Therefore, the trends with respect to crack width should be taken as indicative.

### Implications for design of functionally layered concrete

In regions remote from cracks, the MC durability layer was effective in reducing the depth of carbonation penetration relative to a homogeneous LC beam. In regions adjacent to cracks, the thickness of the durability layer did not appear to have a significant influence on the depth of carbonation $x_{{c,{{max}}}}$ reached in the top layer of concrete but did impact the width of the crack influencing zone. In the specimens with layer thickness of 15 mm and 25 mm, carbonation in the LC mix was visible at the level of the internal steel, as seen in Fig. 17. However, in the 40 mm specimen where the reinforcement is within the durability layer, the carbonation front progressed more slowly through cracks at this level. This suggests that use of a durability layer which fully encases the steel in locations where flexural cracks are likely would be desirable. Such a layer would protect the concrete surrounding the steel from carbonating and facilitating corrosion, whilst enabling the material in the top layer to carbonate at no detriment to the structural capacity of the member. As such, the flexural cracks could present a controlled route for preferential carbonation ingress into the inner layer concrete to sequester carbon dioxide through concrete re-carbonation.

The durability layer thickness is directly proportional to the embodied carbon of the member, with thicker durability layers resulting in overall higher CO$_2$. The relative cement content of the beams is presented in Table 1 as a proxy of CO$_2$ since the cement content dominates the overall footprint. The relative values for the 100 mm $\times$ 100 mm $\times$ 500 mm LC100, MC15LC85, MC25LC75, MC40LC60 and MC100 beams are 75$\%$, 79$\%$, 81$\%$, 85$\%$ and 100$\%$ respectively. Hence, a thicker durability layer encasing the embedded steel has a higher relative cement usage than thinner layers. The thicker 40 mm layer used in MC40LC60 was more practical when casting, since the dimension of the layer was larger compared to the maximum aggregate size, and making larger quantities of the durability mix enabled better utilisation of concrete batch volumes. MC40LC60 had a relative cement content that was still 15$\%$ lower than that of the beam made entirely of MC concrete. However, a full life cycle assessment would need to be conducted to better quantify the environmental benefits of functionally layered concrete designed to provide durability resistance.

A further consideration that impacts the required layer thickness are the variations in the layer thickness that arise due to the deposition process and the properties of the mix layer compositions. It has been shown elsewhere [24, 28] that the layer thickness can vary not only along the length of a specimen but also through the width of the specimen. A better understanding of the possibility of preferential longitudinal carbonation ingress along a layer interface, as noted in MC25LC75.LW, is also required to ensure that the steel remains protected.

## Conclusions

Functionally layered concrete consisting of an outer higher performance durability layer and an inner lower grade bulk layer offers a potential means to reduce the overall embodied CO$_2$ of an element under defined exposure conditions. However, while the carbonation behaviour of functionally layered concrete in the uncracked state has shown promise, there is a lack of understanding about whether flexural cracks that propagate through the durability layer could be detrimental for carbonation ingress. These factors are a concern for reinforced concrete as it could make internal steel vulnerable to corrosion.

An experimental series was undertaken where pre-cracked reinforced beams with durability layers of varying thicknesses (15 mm, 25 mm or 40 mm) were subjected to 4% accelerated carbonation conditions with sustained target crack widths of either 0.15 mm or 0.30 mm. The durability layer concrete had a higher cement content, and thus carbon footprint, than that of the internal concrete. Comparative homogeneous beams consisting of each of the two mixes were also tested. The beam specimens were split after the desired carbonation exposure duration to reveal the carbonation fronts. The fronts were extracted using digital image processing techniques which were shown to be an effective means to identify the continuous, and tortuous, carbonation profile in cracked and uncracked regions.

In regions remote from cracks it appeared that the carbonation ingress in the uncracked regions of the homogeneous and layered beams carbonation was not dependent on the applied compressive or tensile stress for the stress levels used here. In the layered beams, the uncracked free face carbonation of the top layer was analogous to that of the top layer concrete whereas the uncracked bottom face regions exhibited carbon depths akin to that of the uncracked durability layer concrete. So in uncracked regions the durability layer provided a protective barrier.

In regions adjacent to the cracks, local extremes in carbonation depth were detected around crack locations, indicating that the presence of cracks accelerated carbonation in these regions. A greater crack width led to a larger maximum penetration depth. Carbonation ingress was observed through crack faces that extended into the interior layer concrete of the layered specimens. Implementing a durability layer of higher cement mix which encased the reinforcement helped reduce the extent of carbonation around the reinforcement but with a lower overall embodied carbon. An added benefit would be that carbonation of the lower-cement top layer mix could sequester additional CO$_2$ from the environment.

## Data Availability

The authors confirm that the data supporting the findings of this study are available within the article.
